# A Case of Primary Hepatic Lymphoma and Related Literature Review

**DOI:** 10.1155/2016/6764121

**Published:** 2016-06-14

**Authors:** Yonghua Liu, Jinhong Jiang, Qinli Wu, Qiaolei Zhang, Yehui Xu, Zhigang Qu, Guangli Ma, Xiaoqiu Wang, Xiaoli Wang, Weimei Jin, Bingmu Fang

**Affiliations:** Department of Hematology, Sixth Affiliated Hospital of Wenzhou Medical University, Lishui City, Zhejiang 323000, China

## Abstract

*Objective*. Primary hepatic lymphoma is a rare disease. And the clinical manifestations of this disease are nonspecific. The objective of this paper is to improve clinicians' understanding of this disease.* Methods*. We analyzed the clinical characteristics of a case of primary hepatic lymphoma in association with hepatitis B virus infection and reviewed the literature.* Conclusion*. The clinical manifestations of primary hepatic lymphoma are nonspecific. And it is easily misdiagnosed. Postoperative radiotherapy of patients with early stage was previously speculated to achieve favorable improvement. The application of targeted therapeutic drugs, chemotherapy, or combined local radiotherapy has become the first-line treatment strategy.

## 1. Background

Primary hepatic lymphoma (PHL) refers to tumor confined in the liver at the early stage of lymphoma without infiltration of other locations. PHL is a rare disease, with an incidence of only 0.1% for malignant liver tumors. This disease also accounts for 0.4% of all primary extranodal lymphoma and 0.016% of all non-Hodgkin's lymphoma [1]. PHL of diffuse large B-cell lymphoma (PHL-DLBCL) is more infrequent. The clinical manifestations of PHL-DLBCL are nonspecific. Hence, the condition is difficult to distinguish from primary liver cancer, liver metastases, granulomatous pseudotumor, and other diseases and is thus easily misdiagnosed. To improve clinicians' understanding of this disease, the diagnosis and treatment course of a case of PHL in our hospital were discussed in detail.

## 2. Case Introduction

A 61-year-old male was admitted because of “fatigue and abdominal distension for over 50 days.” The patient presented with a 6 kg weight loss (12%) and experienced fatigue and abdominal distension from an unknown cause since 50 days prior to admission. The patient showed no signs of skin pruritus or yellow discoloration of the skin or eyes. Despite his symptoms, the patient did not initially seek medical consultation. He was then admitted in a local hospital from June 6 to June 12, 2014 because of fever. The patient suffered from chills and sweating, and his body temperature fluctuated at 37.4–38.9°C. He was diagnosed with “cholecystitis, fatty liver, hepatomegaly, and fever (unknown).” He was then treated with cefoperazone/sulbactam sodium for the inflammation, pantoprazole to relieve hyperacidity, and polyene phosphatidylcholine to protect the liver, all of which afforded the patient with no improvement of condition. The patient sought consultation in one of the top three local hospitals on June 19. His signs and symptoms of fatigue, abdominal distension, and yellow urine aggravated, along with the decrease in urine amount. At the time, the patient presented with a urine output of less than 500 mL/24 h, with concurrent skin pruritus, icteric skin and sclerae, occasional cough, and severe bilateral lower extremities edema. He also experienced slight chest tightness and hence was admitted to the hospital.

**Figure 1:**
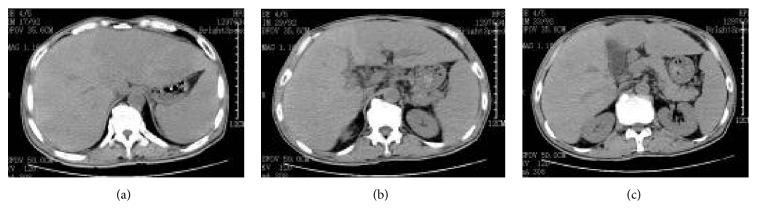
Epigastrium CT before treatment. Note: the liver outline was significantly more prominent. The liver edge was less smooth. Liver parenchymal density was uneven. Patchy low-density shadows were observed, especially in the left live. Multiple quasicircular low-density foci appeared in the right lobe of the liver. The spleen was enlarged to about seven rib units. The density was uniform.

**Figure 2:**
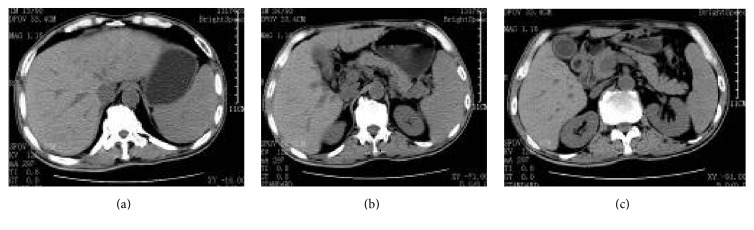
Epigastrium CT after treatment. Note: the liver outline was significantly more prominent. The liver edge was less smooth. Liver parenchymal density was uniform. Multiple quasicircular low-density foci were found in the right lobe of the liver, but no intensified low-density focus was noted. The largest diameter was about 5.5 mm. The spleen was enlarged to approximately seven rib units. The density was uniform. Compared with the CT findings on July 11, the liver density increased and the density was more uniform.

**Figure 3:**
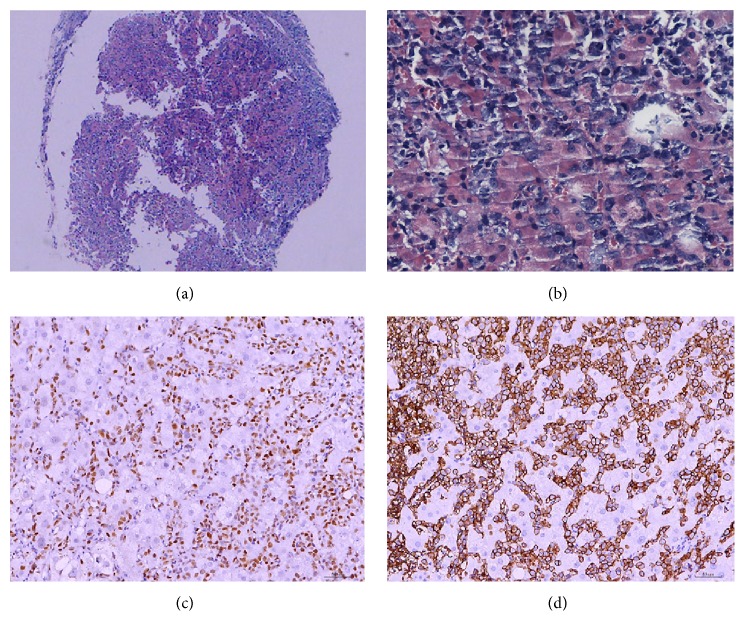
Liver biopsy pathology and histochemistry (hematoxylin-eosin staining,). (a) and (b) Heteromorphic lymphocytes were observed in the hepatic sinusoid and portal area. The nuclei were round or oval. The cells presented with one or multiple nucleoli. The nuclear mitotic figure was common, low, and high magnifications (×4, ×40, resp.). (c) BCL-6 was diffusely strongly positive. (d) CD20 was diffusely strongly positive.

Upon admission, routine blood examination obtained the following results: white blood cell count, 9.3 × 10^9^/L; neutrophil absolute value, 4.9 × 10^9^/L; hemoglobin, 128 g/L; and platelet count, 171 × 10^9^/L. Meanwhile, analysis of serum biochemistry revealed the following levels: albumin, 28 g/L; ALT, 128 U/L; AST, 109 U/L; total bilirubin, 12.5 *μ*mol/L; direct bilirubin, 2.7 *μ*mol/L; indirect bilirubin, 125 *μ*mol/L; hydroxybutyrate dehydrogenase, 1093 U/L; lactate dehydrogenase, 1387 U/L; and Total Bile Acids (TBA), 2.461 *μ*mol/L. The liver fibrosis index is as follows: hyaluronic acid, 1162.2 *μ*g/L; type IV collagen, 279.4 *μ*g/L; type III collagen, 659.8 *μ*g/L; and laminin, 149 *μ*g/L. The blood ammonia level was 365 *μ*mol/L, whereas that of C-reactive protein was 136.4 mg/L. Coomb's test was negative. Hepatitis B screening tested positive for three components: HBV-DNA, 3.1*E* + 02 copy/mL. Chest computed tomography (CT) scan revealed the following findings: (1) small fibrotic foci at the middle lobe of the right lung and lingular segment of the superior lobe of the left lung and (2) emphysema in the superior lobe of the right lung and calcified foci at the superior lobe of the left lung. No swollen lymph nodes were found by ultrasound on the bilateral neck, supraclavicular, subaxillary, and inguinal regions. Abdominal CT (July 11, 2014) displayed (1) hepatosplenomegaly, ascites, patchy low-density shadows in the liver (especially in the left liver), and multiple low-density lesions in the right lobe of liver; (2) gallbladder wall thickening; and (3) prostate calcification (Figure 1). The patient was treated with entecavir dispersible tablet 0.5 mg qd, oral administration of antiviral drugs, and drugs for liver protection and diuresis. The liver progressively enlarged, with a significant increase in bilirubin. The patient underwent liver biopsy. Pathological examination showed the following results: heteromorphic lymphocytes were observed in the hepatic sinusoid and portal area. The nuclei were round or oval. The cells displayed single or multiple nucleoli. The nuclear mitotic figure was common. For immunohistochemistry, LCA, CD20, and BCL-6 were diffusely strongly positive; CD79a and Mum-1 were locally weakly positive; the Ki-67 labeling index was about 60%; and CK, CD68, CD34, Hepaar-1, CD3, CD43, CD10, and ALK were negative; HBs-Ag and HBc-Ag were negative (Figure 3). Bone marrow morphology and biopsy showed no signs of lymphoma infiltration. On the basis of clinical, laboratory, and pathological examinations, the patient was diagnosed with primary hepatic non-Hodgkin's lymphoma staged IVA (DLBCL, non-GCB), chronic viral hepatitis (B), and decompensated cirrhosis. CHOP (cyclophosphamide, doxorubicin, vincristine, and prednisone) scheme chemotherapy was then initiated on July 17 with the following agents: IFO, 2 g (Days 1 and 8); THP, 60 mg (Day 1); DXM, 10 mg (Days 1 to 5); and VDS, 4 mg (Days 1, 8, and 15). The chemotherapeutic course was uneventful. The liver significantly shrunk in three days after chemotherapy was completed. The CT review after the first chemotherapy (August 11) showed that the liver significantly decreased in size, and liver density became uniform (Figure 2). These findings suggest that the treatment was satisfactory and achieved partial remission. The R-CHOP scheme was adopted for six cycles (CHOP with rituximab injection, 500 mg [Day 0]); the other drugs included were the same as those mentioned above). No new lesion was noted, and the patient has lived for nearly 2 years till now of disease-free-survival.

## 3. Literature Review

Primary hepatic lymphoma (PHL) refers to the lesion only confined to the liver at the early stage of lymphoma. Infiltrations of lymph nodes, spleen, bone marrow, and other organs must be excluded at the onset. The patient's clinical characteristics include abdominal distension, fatigue, abnormal liver function, and progressive hepatomegaly. This patient was diagnosed with non-Hodgkin's lymphoma by liver biopsy. The pathological type was DLBCL. The involvement of other locations was excluded by superficial lymph node examination, bone marrow morphology and biopsy, and radiologic examinations. The diagnosis of PHL-DLBCL was confirmed.

### 3.1. Epidemiological Analysis of PHL

PHL may occur at any age but more commonly in males of about 50 years old. The male-to-female ratio for the disease incidence is about 3 : 1 [2]. Liver involvement accounts for about 15%–17% of non-Hodgkin's lymphoma. However, primary liver non-Hodgkin's lymphoma is extremely rare, accounting for 0.4% of all primary extranodal lymphoma and 0.016% of all cases of non-Hodgkin's lymphoma. The etiology of PHL is uncertain and may include virus infection (HIV, AIDS, HBV, HCV, and EBV), autoimmune diseases, and immune inhibitor application [3]. Its mechanism potentially involves T lymphocyte loss of inherent immune surveillance and function after viral infection or application of immunosuppressive agents. This occurrence may result in unrestrained lymphopoiesis, thereby forming lymphoma.

### 3.2. Clinical Manifestations of PHL

The clinical manifestations of PHL are nonspecific. Most patients seek consultation because of fatigue, loss of appetite, night sweats, low-grade fever, and weight reduction. Some patients show hepatomegaly, abdominal pain, or liver function abnormalities. Disease progression is rapid. Several patients suffer from hepatic encephalopathy early, resulting in coma and even death [2, 4]. In our case, the onset of disease was acute. The patient experienced abdominal distension and fatigue and sustained low fever, hepatomegaly, and progressive increase of bilirubin, which were rapidly relieved by treatment. The patient's clinical characteristics were consistent with that of primary hepatic non-Hodgkin's lymphoma. For laboratory indices, most PHL patients show elevated ALT and AST levels by two to three times of the normal value, which is also significantly increased with the increase in bilirubin and HDL. However, AFP and CEA levels are usually normal, in contrast to those in primary liver cancer and liver metastasis.

A common radiologic finding in the disease is liver space-occupying lesions. Most patients show single lesions, as well as multiple space-occupying lesions. Some patients sustain diffuse liver lesions, displayed in radiologic examination as hepatomegaly without liver space-occupying lesion. PHL radiologic manifestations often involve a nonuniform liver texture. The central mass mainly shows low-density shadows in CT and sometimes multilobar or patchy low-density shadows, which are peripherally intensified after enhancement. These low-density radiologic findings differ from those of other solid hepatic tumors. This result may be explained by the lesser vascularization of the lymphoma compared with other tumors, resulting in tissue necrosis during the disease process. MRI revealed high-density T1 and high-signal T2. The radiologic performance of the patient showed that the liver was obviously enlarged and the liver texture was not uniform, combined with patchy low-density shadows, which was consistent with literature reports [5, 6].

Most hepatic lymphomas are derived from B-lymphocyte cells, whereas the minority originates from T lymphocytes. The diagnosis should depend on liver tissue biopsy, including immunohistochemistry. PHL patients with liver function abnormality, hepatomegaly, or space-occupying lesions but with normal AFP and CEA must be suspected of liver lymphoma. Laparoscopic examination or percutaneous liver biopsy is highly necessary. However, tissue necrosis is often associated with liver lymphoma; hence, fine needle aspiration biopsy may provide false negative results. PHL was often misdiagnosed as other conditions, such as embryonic sarcoma, inflammatory pseudotumor, or granulomatous hepatitis. Occasionally, immunohistochemistry still cannot sufficiently obtain a satisfactory diagnosis. Cellular immunology, gene rearrangement, cell genetics, and molecular biology related to PHL should be explored to assist diagnosis and determine prognosis.

### 3.3. PHL Treatment

Postoperative radiotherapy of patients with early PHL was previously speculated to achieve favorable improvement [7]. However, with the continuous development of chemotherapeutic regimens, especially the application of targeted therapeutic drugs, chemotherapy or combined local radiotherapy has become the first-line treatment of PHL. The chemotherapy regimen often applied is mainly based on CHOP [8]. Our patient was diagnosed with PHL-DLBCL. The single-course treatment with CHOP combined with rituximab was significant. The liver was significantly reduced in size, and the low-density lesions were significantly decreased. In a previous study, the follow-up visits of 24 PHL cases after 20 years by the Anderson Cancer Center in the United States revealed a complete remission rate of 85% through chemotherapy and an event-free five-year survival of 70% [9].

PHL is an extremely rare condition in clinical practice. Moreover, PHL-DLBCL is rarely reported worldwide. A considerable number of prospective, randomized controlled clinical trials are lacking and must be further conducted.
